# Natural Antioxidants Enhance Post-Thaw Sperm Quality and Fertilization Performance in Javaen Barb (*Systomus rubripinnis*)

**DOI:** 10.3390/antiox15091135

**Published:** 2026-09-08

**Authors:** Irfan Zidni, Abinawanto Abinawanto, Akhmad Taufiq Mukti, Aisyah Aisyah, Suci Lestari, Yuli Andriani, Iskandar Iskandar, Roffi Grandiosa, Atikah Nurhayati, Pringgo Kusuma Dwi Noor Yadi Putra, Mochammad Ikhsan Cahya Utama, Elva Yenizar Anggraeni

**Affiliations:** 1 Department of Fisheries, Faculty of Fisheries and Marine Science, Universitas Padjadjaran, Sumedang Regency 45363, West Java, Indonesia; aisyah@unpad.ac.id (A.A.); yuli.andriani@unpad.ac.id (Y.A.); iskandar@unpad.ac.id (I.I.); roffi.grandiosa@unpad.ac.id (R.G.); atikah.nurhayati@unpad.ac.id (A.N.); pringgo.kusuma@unpad.ac.id (P.K.D.N.Y.P.); mochhamad.ikhsan@unpad.ac.id (M.I.C.U.); elva21001@mail.unpad.ac.id (E.Y.A.); 2 Department of Biology, Faculty of Mathematics and Natural Sciences, Universitas Indonesia, Depok 16424, West Java, Indonesia; abinawanto.ms@sci.ui.ac.id; 3 Department of Aquaculture, Faculty of Fisheries and Marine, Universitas Airlangga, Jl. Dr. Ir. Soekarno, Surabaya 60115, East Java, Indonesia; akhmad-t-m@fpk.unair.ac.id; 4 Department of Biology Education, Faculty of Teacher Training and Education, Universitas Muhammadiyah Prof. Dr. Hamka, East Jakarta 13830, Jakarta, Indonesia; suci.lestari@uhamka.ac.id

**Keywords:** natural antioxidant, sperm cryopreservation, germplasm conservation, biodiversity conservation, aquatic genetic resources, sustainable aquaculture, *Systomus rubripinnis*, Javaen barb

## Abstract

The freeze–thaw process in sperm cryopreservation generates reactive oxygen species (ROS) that impair motility, viability, and fertilizing capacity in fish, including the Javaen barb (*Systomus rubripinnis*), an endemic Indonesian cyprinid of conservation concern. Cryopreservation underpins gene bank development for aquatic genetic resources and supports biodiversity conservation alongside hatchery-based stock enhancement. This study evaluated five natural antioxidants: basil leaf extract (BLE), noni fruit extract (NFE), moringa leaf extract (MLE), sambiloto leaf extract (SLE), and honey–egg yolk (HEY). Each antioxidant was supplemented into the Kurokura’s cryopreservation extender across a graded concentration series (BLE 1–4%, NFE and MLE 0.5–1.5%, SLE 6–9%, and HEY 1–2% with a fixed 5% egg yolk) and compared with an antioxidant-free control. Radical scavenging capacity was measured by DPPH assay (IC_50_: BLE 134.06, MLE 297.04, NFE 422.86, SLE 3010.46 ppm), and post-thaw motility, duration of motility, viability, abnormality, fertilization rate, and ATP bioluminescence were assessed. The most effective doses tested were doses of 1% for BLE, NFE, and MLE, 1.25% for HEY, and 7% for SLE. BLE at 1% gave the best overall profile (motility 85.00 ± 0.88%; duration 7.14 ± 0.38 min; viability 82.00 ± 1.86%; abnormality 18.26 ± 0.16%), significantly exceeding the control (65.00 ± 2.31%; 4.56 ± 0.33 min; 68.00 ± 2.42%; 23.54 ± 0.28%; *p* < 0.05), whereas SLE sustained the longest motility. All extracts supported fertilization rates above 82% versus 70.08 ± 3.82% in the control, and 1% BLE preserved post-thaw luminescence equivalent to fresh sperm (165,217 vs. 164,087 RLU), interpreted comparatively. These locally available, plant-derived antioxidants offer a low-cost route to germplasm conservation of Javaen barb, strengthening conservation hatchery and broodstock management, sustainable aquaculture production, and food security for inland fisheries communities.

## 1. Introduction

The Javaen barb (*Systomus rubripinnis*), locally known in West Java as “Beureum Panon”, meaning “red-eyed” in Sundanese, is a small-bodied freshwater cyprinid fish endemic to the river systems of Java, Indonesia. This species inhabits fast-flowing, clear-water streams with gravel and rocky substrates, primarily in the upper reaches of river catchments across West Java. *Systomus rubripinnis* holds dual ecological and socioeconomic significance: it serves as an ecological indicator of pristine freshwater ecosystems and is widely valued in the ornamental fish trade, as well as in small-scale local fisheries deeply embedded in the cultural traditions of West Javanese communities [1]. Unlike widely cultured cyprinids, the Javaen barb is not an established commercial commodity; although it is occasionally taken in small-scale local fisheries, its wild populations have declined sharply and, in parts of Java, have been extirpated, so the motivation for safeguarding its germplasm is ecological and conservation-driven rather than commercial. Morphologically, adult *S. rubripinnis* is distinguished by its vivid red iris, compact body form, and bright coloration, making it one of the more aesthetically notable endemic cyprinids of the Sundanic bioregion [2].

Despite its ecological and cultural importance, *S. rubripinnis* faces escalating conservation threats. Habitat degradation driven by intensive agriculture, land-use conversion, sedimentation, and domestic water pollution has severely contracted the species’ natural range and reduced its population density in native river systems. Overexploitation for the ornamental fish trade has further compounded the decline. *S. rubripinnis* is currently assessed as Vulnerable on the IUCN Red List of Threatened Species [3], reflecting the significant risk of further population reduction without targeted conservation intervention. This situation mirrors the broader global crisis of freshwater biodiversity, wherein the 2022 WWF Living Planet Report documented an 83% average decline in freshwater vertebrate population indices since 1970 [4], driven primarily by habitat loss, overextraction, and the disruption of aquatic connectivity. The urgency of developing effective ex situ conservation strategies for endemic freshwater species such as *S. rubripinnis* is therefore considerable.

Sperm cryopreservation has emerged as one of the most effective and scientifically established ex situ biotechnologies for the preservation of fish genetic resources and the facilitation of assisted reproduction in both conservation and aquaculture contexts [5,6]. By enabling the long-term storage of spermatozoa at cryogenic temperatures (−196 °C), this technology circumvents the temporal and spatial constraints inherent to natural spawning cycles, facilitating year-round artificial fertilization, selective breeding, and the establishment of cryogenic gene banks for endangered species [7,8]. The application of sperm cryopreservation in freshwater cyprinid fish has expanded significantly in recent decades, with successful protocols developed for endangered species, including *Microphysogobio rapidus* in South Korea [9], *Tor tor* [10], *Puntius sarana* [11], *Neolissochilus soroides* [12], *Cyprinus carpio* L. [13], lake minnows (*Eupallasella percnurus*) [14], rare minnow (*Gobiocypris rarus*) [15], and various European cyprinids [16]. For *S. rubripinnis*, the development of a robust cryopreservation protocol would directly underpin the establishment of a national genetic resource bank for this species, supporting both hatchery-based production and in situ population recovery.

A central challenge in fish sperm cryopreservation is the induction of oxidative stress during the freeze–thaw cycle [17]. The cryogenic process disrupts mitochondrial electron transport chain activity, generating excess reactive oxygen species (ROS) that initiate the lipid peroxidation of sperm plasma membranes, which are particularly vulnerable due to their high content of polyunsaturated fatty acids (PUFAs), while simultaneously inducing DNA strand breaks and mitochondrial dysfunction [7,18]. The resultant oxidative damage manifests as reduced post-thaw sperm motility, shortened motility duration, impaired membrane integrity, increased morphological abnormality, and, ultimately, reduced fertilization competence [8,19]. Antioxidants, which function by scavenging free radicals, quenching singlet oxygen, and upregulating endogenous antioxidant enzyme systems (superoxide dismutase, catalase, glutathione peroxidase), represent the primary pharmacological strategy for mitigating cryodamage in spermatozoa [6]. Synthetic antioxidants such as butylated hydroxytoluene (BHT), ascorbic acid, α-tocopherol, and catalase have demonstrated variable protective effects on fish sperm post-thaw quality across multiple species [19,20].

Growing concerns over the biocompatibility and environmental safety of synthetic antioxidants have directed research attention toward natural plant-derived alternatives with equivalent or superior antioxidant efficacy [6]. Among natural antioxidants evaluated in fish sperm cryopreservation, polyphenolic compounds such as quercetin, curcumin, resveratrol, and epigallocatechin gallate (EGCG) have been reported to improve post-thaw motility, viability, and DNA integrity in species, including common carp (*Cyprinus carpio*), rainbow trout (*Oncorhynchus mykiss*), and Nile tilapia (*Oreochromis niloticus*) [21]. In spotted halibut (*Verasper variegatus*), supplementation of cryopreservation media with natural antioxidants significantly improved post-thaw sperm motility and viability compared to unsupplemented controls, confirming the practical benefit of antioxidant addition in marine teleost cryopreservation [22]. Honey-derived products and egg yolk, both of which possess complex bioactive matrices with antioxidant and membrane-stabilizing properties, have been incorporated into mammalian semen extenders with documented cryoprotective benefits [23]. Similarly, various Indonesian medicinal plants, known locally as *tanaman obat*, have yielded bioactive extracts with potent in vitro antioxidant activity, as assessed by the DPPH radical scavenging assay, yet their direct application to fish sperm cryopreservation has not been systematically investigated.

Several natural antioxidant candidates with well-characterized bioactivity in mammalian reproductive studies have, to our knowledge, not been evaluated in the context of fish sperm cryopreservation. Basil (*Ocimum basilicum*) leaf extract rich in eugenol, linalool, quercetin, and rosmarinic acid has been shown to support spermatogenesis and reduce testicular oxidative damage in rats and to improve semen quality parameters in rams [24,25], but its application in fish has not been reported. Noni fruit (*Morinda citrifolia*) extract, containing iridoids (asperulosidic acid, deacetylasperulosidic acid), flavonoids (kaempferol, rutin), and scopoletin, is known to upregulate endogenous antioxidant enzymes in mammalian systems [26], but has not been tested in fish sperm cryopreservation. *Moringa oleifera* leaf extract, which has demonstrated significant improvements in post-thaw sperm motility, membrane integrity, and reduced lipid peroxidation in ram and buck spermatozoa [27,28,29], remains unexplored in teleost fish. Honey, studied as an antioxidant additive in rabbit and bovine sperm extenders [23], and *Andrographis paniculata* (sambiloto), whose primary constituent andrographolide modulates mitochondrial antioxidant pathways and has immunomodulatory effects [30], similarly lack documentation in fish sperm cryopreservation. These knowledge gaps represent a significant frontier in applied fish reproductive biotechnology, particularly for tropical endemic species where locally sourced natural products offer practical and sustainable advantages.

This study therefore aimed to evaluate the comparative effects of five plant-derived and natural antioxidant supplements, BLE, NFE, MLE, SLE, and HEY, on post-thaw sperm quality parameters and fertilization performance in Javaen Barb (*Systomus rubripinnis*), and to determine the most effective supplementation dose for each antioxidant type. Intracellular ATP content was additionally quantified to assess sperm metabolic status following cryopreservation under each antioxidant regimen. The results are expected to provide a scientific basis for the development of improved, nature-based cryopreservation protocols for Javaen Barb hatcheries and to contribute to the broader toolkit of ex situ conservation biotechnologies for endemic freshwater fish species in Indonesia.

## 2. Materials and Methods

### 2.1. Ethics Statement

This research was approved by the Ethics Committee of Universitas Padjadjaran, under reference/approval number 492/UN6.KEP/EC/2026. All experimental procedures involving fish were conducted in accordance with institutional and national guidelines for the care and use of animals in research.

### 2.2. Plant Material and Extract Preparation

Leaves of basil (*Ocimum basilicum*; BLE), fruit of noni (*Morinda citrifolia*; NFE), leaves of moringa (*Moringa oleifera*; MLE), and leaves of sambiloto (*Andrographis paniculata*; SLE) were used as plant material. Fresh material was collected, cleaned, cut into small pieces, and dried into simplicia. Crude extracts were prepared by maceration following the standard operating procedure of the Central Laboratory, Universitas Padjadjaran (document no. LS/PP/BA.01-000/00), which is based on the Indonesian Ministry of Health general standard for medicinal-plant extracts [Depkes RI, 2000]. Each dried sample was macerated in ethanol at a sample-to-solvent ratio of 1:5–1:10 (*w*/*v*) in a vessel sealed with aluminum foil to limit solvent evaporation, and left at room temperature for 24 h with occasional stirring. The mixture was then filtered through filter paper; the residue was re-macerated with fresh solvent, and maceration followed by filtration was repeated stepwise up to 3 × 24 h.

### 2.3. Broodstock Management

Javaen barb (*S. rubripinnis*) used in this study were maintained in the Wet Laboratory Ciparanje, Jatinangor, Sumedang, West Java, Indonesia, during the spawning season (January to April). Fifteen adult males (mean weight: 35.75 ± 0.49 g; mean total length: 19.33 ± 0.23 cm) were distributed in broodstock tanks equipped with separate recirculation systems, with water quality conditions of temperatures of 25–30 °C, dissolved oxygen of >5 mg/L, and pH of 6.5–8.5. Each day, the Javaen barb were fed twice a day with a commercial floatable feed (Hiprovite 781-1 PT Central Pangan Pertiwi Tbk, Karawang, West Java, Indonesia).

### 2.4. Sperm Collection

Spermatogenesis was stimulated by intramuscular injection of Ovaprime^®^ (Syndel, Nanaimo, CA, Canada) at 0.3 mL/kg body weight. Prior to sampling, fish were anesthetized with Arowana Stabilizer (Ocean Free, Singapore) at 1 ppm (1 mg/L). Semen was collected by gentle abdominal stripping using sterile 1.0 mL syringes, with care taken to avoid contamination by urine, blood, or excrement. The sperm samples were kept cold (4 °C) and delivered to the lab. For this investigation, samples of spermatozoa with an overall motility of greater than 80% were collected. To obtain sufficient sample volume for all treatments and to minimize inter-individual variability, the qualifying semen from the fifteen males was combined into a single homogeneous pooled sample, from which all antioxidant treatments and their three replicates (*n* = 3) were drawn. Sperm volume per individual ranged from 0.20 to 0.59 mL, with a concentration of approximately 285,000,000–384,000,000 cells/mL.

### 2.5. Sperm Cryopreservation

Sperm cryopreservation was performed using a medium containing cryoprotectants 10% methanol in Kurokura’s Extender (NaCl 61.22 mmol/L, KCl 134.14 mmol/L, CaCl_2_ 1.99 mmol/L, MgCl_2_ 0.84 mmol/L, and NaHCO_3_ 2.39 mmol/L; pH 8.0), supplemented with several natural antioxidants including BLE (1%, 2%, 3%, 4%), NFE (0.5%, 0.75%, 1%, 1.25%, 1.5%), MLE (0.5%, 0.75%, 1%, 1.25%, 1.5%), SLE (6%, 7%, 8%, 9%), and HEY with the addition of 5% egg yolk at each concentration (1%, 1.25%, 1.5%, 1.75%, 2%) at the tested concentrations. These concentration ranges were established from preliminary dose-finding trials and were scaled to the radical scavenging potency of each extract determined by the DPPH assay (Table 1): extracts with strong scavenging capacity and low IC_50_ values, such as BLE (134.06 ppm), were applied at lower inclusion levels (1–4%), whereas the markedly weaker SLE (IC_50_ = 3010.46 ppm) required proportionally higher concentrations (6–9%) to achieve comparable functional antioxidant activity. The selected ranges were also consistent with effective doses previously reported for plant-derived antioxidants in fish and mammalian sperm cryopreservation. Fresh sperm was mixed with the cryopreservation medium at a ratio of 1:10 (*v*/*v*) under sterile conditions. The extract percentages denote the concentration in the extender prior to the addition of semen; because semen and supplemented extender were combined at 1:10 (*v*/*v*, semen:extender), the nominal values were diluted by approximately 9% in the final freezing medium and loaded into 0.25 mL straws (Sterile Classical PETG Sperm Straw, L’Aigle, France), and sealed with sealing powder (Reproduction Provisions, Walworth, WI, USA). The freezing protocol followed Lee et al. [31], with the first cooling performed by placing the straws in a floating device composed of a tray (8.5 cm wide and 20 cm long) within an expanded polystyrene box at a height of 3 cm above nitrogen vapor for 2 min, and a lid was utilized throughout the initial chilling process to keep the temperature steady. In the second cooling, the straws were immersed in liquid nitrogen (−196 °C). Cryopreserved sperm samples were stored in liquid nitrogen for 48 h. The >80% motility criterion applied to fresh semen at collection, prior to freezing. The control group consisted of sperm from the same pooled sample cryopreserved in the antioxidant-free base extender and assessed post-thaw; its lower post-thaw motility (65.00%) therefore reflects freeze–thaw damage in the absence of antioxidant protection, against which the supplemented treatments were compared. All treatments were performed in triplicate. Thawing was conducted in distilled water at 25 °C prior to fertilization assessment.

### 2.6. Antioxidant Activity Assay (DPPH)

The antioxidant activity of all extracts was evaluated using the DPPH (2,2-diphenyl-1-picrylhydrazyl) radical scavenging assay prior to the cryopreservation experiment. Each extract solution was mixed with DPPH reagent and incubated in the dark at room temperature for 30 min. Absorbance was measured spectrophotometrically at 517 nm, and the percentage of radical scavenging activity was calculated. IC_50_ values (concentration required to inhibit 50% of DPPH radicals) were determined from dose–response curves and used to characterize relative antioxidant potency. The individual bioactive constituents of the extracts were not quantified in the present study; the compound classes referred to hereafter were inferred from the DPPH radical scavenging activity and from phytochemical profiles previously characterized for each species in the literature. Comprehensive phytochemical profiling (e.g., LC-MS/MS and HPLC) to identify and quantify the specific compounds responsible for the observed cryoprotective effects is planned for subsequent work.

### 2.7. Sperm Motility Evaluation

The sperm motility was calculated as the proportion of motile sperm relative to total sperm, evaluated under 200× magnification (MSC-V208, Infitek, Shanghai, China). Activation was performed using filtered freshwater (1:100 ratio) at 25 °C, and motility parameters were recorded within 30 s of dilution. The microscope was connected to a computer equipped with an image driving software (Win 10 Pad software).

### 2.8. Duration of Motility

The duration of motility was evaluated as the time elapsed from the initiation to the cessation of spermatozoa movement, and was expressed in minutes. Frozen semen straws were thawed in a water bath at 25 °C for 15 s. Immediately after thawing, a drop of semen was placed on a clean, pre-warmed glass slide, covered with a coverslip, and examined under a light microscope at ×400 magnification fitted with a stage maintained at 25 °C. The progressive movement of spermatozoa was recorded by video documentation, from which the duration of motility was measured as the interval between the start of observation and the moment at which no spermatozoa movement could be observed. Evaluations were carried out in a 48 h storage period. Each treatment was assessed in three replicates (*n* = 3). At each time point, motility (%), duration of motility (min), and viability (%) were evaluated successively for the same sample.

### 2.9. Sperm Viability Evaluation

Sperm viability was assessed using the Cell Counting Kit-8 (CCK-8) colorimetric assay (Dojindo Laboratories, Kumamoto, Japan). CCK-8 reagent was added to sperm suspension at a 1:10 volume ratio, and 100 µL aliquots were transferred to 96-well plates. Following incubation for 1–4 h until orange color development, absorbance was measured at 450 nm using a Spectra Max 190 microplate reader (Molecular Devices, San Jose, CA, USA). Formazan production by cellular dehydrogenases was used as an indicator of viable cell proportion.

### 2.10. ATP Quantification

ATP content in sperm was quantified using the CellTiter-Glo™ 2.0 Luminescent Cell Viability Assay (Promeg, Madison, WI, USA) following the manufacturer’s protocol. Sperm samples were prepared at a density of 101.6 × 10^6^ cells/mL and transferred (100 µL) into white opaque 96-well microplates. An equal volume (100 µL) of CellTiter-Glo^®^ reagent was added, followed by gentle orbital mixing for 2 min and incubation for 10 min at room temperature to stabilize luminescent signal. Luminescence was measured using an Infinite 200 PRO microplate reader (Tecan Group, Männedorf, Switzerland) and expressed as relative luminescence units (RLU). All measurements were performed in triplicate. Results are expressed as relative light units (RLU). Because RLU is an arbitrary unit specific to the instrument and reagent used, values were used only for relative comparisons between treatment groups under identical assay conditions.

### 2.11. Fertilization Experiment

The fertilization experiment was conducted at the Wet Laboratory Ciparanje, Jatinangor, Sumedang, West Java. Fresh eggs were collected from females of *S. rubripinnis* (*n* = 7, mean length 19.33 ± 0.23 cm and mean weight 35.75 ± 0.49 g). Eggs stripped from the seven females were pooled into a single homogeneous batch and thoroughly mixed; equal aliquots of this pooled batch were assigned to every treatment and to each of the three replicates, so that all treatments were fertilized with eggs of identical composition and no treatment was tied to an individual female. The ratio of sperm to eggs was 1:5 (*v*/*v*, 0.10–0.50 mL), and fertilization proceeded for 2 min followed by three washes with fresh water. A total of 250 fertilized eggs per replicate were distributed in 15 cm Petri dishes and incubated at 25 °C. Fertilization rates were determined by counting the number of eggs that reached the eight-cell stage at 1 h 30 min post-fertilization under 40× magnification (CH30, Olympus, Japan). Each treatment was replicated three times.

### 2.12. Statistical Analysis

Sperm quality parameters are reported as means ± standard errors (SEs). Data were standardized by arcsine square-root transformation. Statistical analysis was performed in SPSS v. 23. One-way analysis of variance (ANOVA) and Duncan’s multiple range test were conducted using a significance threshold of *p* < 0.05.

## 3. Results

### 3.1. Antioxidant Activity (IC_50_) of the Natural Extracts

The DPPH radical scavenging activity and corresponding IC_50_ values of the evaluated plant extracts are presented in Table 1. Honey–egg yolk (HEY) was excluded from the DPPH assay as it represents a complex, non-plant biological matrix rather than a pure antioxidant extract. Among the four evaluated plant extracts, basil leaf extract (BLE) exhibited the most potent antioxidant activity, yielding the lowest mean IC_50_ value of 134.06 ppm (Replicate 1: 134.92 ppm; Replicate 2: 133.21 ppm), structurally classifying its capacity as moderate-to-strong depending on the standard threshold utilized. Moringa leaf extract (MLE) and noni fruit extract (NFE) demonstrated intermediate scavenging capabilities, with mean IC_50_ values of 297.04 ppm and 422.86 ppm, respectively. Conversely, sambiloto leaf extract (SLE) exhibited exceptionally weak antioxidant activity, resulting in the highest mean IC_50_ value of 3010.46 ppm (Replicate 1: 3011.24 ppm; Replicate 2: 3009.68 ppm). This indicates that a substantially higher concentration of SLE is required to achieve an equivalent 50% radical-neutralizing effect compared to the other botanical treatments.

### 3.2. Post-Thaw Sperm Quality Under Basil Leaf Extract (BLE) Supplementation

**Figure 1:**
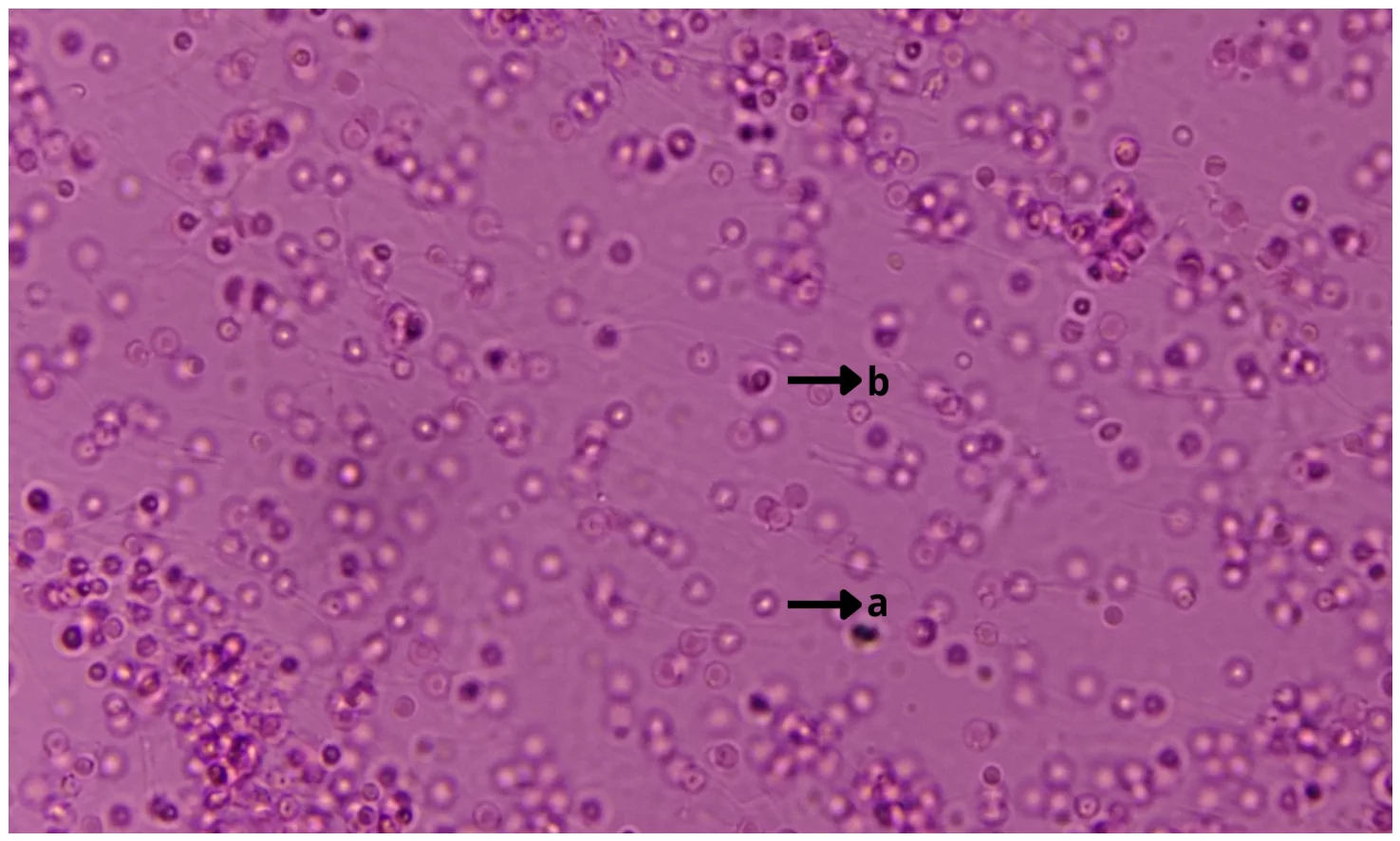
Spermatozoa of Javaen barb (*Systomus rubripinnis*) cryopreserved with basil leaf extract (BLE), stained with eosin-Y for viability assessment. Label (a) indicates viable spermatozoa and label (b) non-viable spermatozoa, observed under a light microscope (CH30, Olympus, Tokyo, Japan) at 40× objective magnification (400× total).

**Figure 2:**
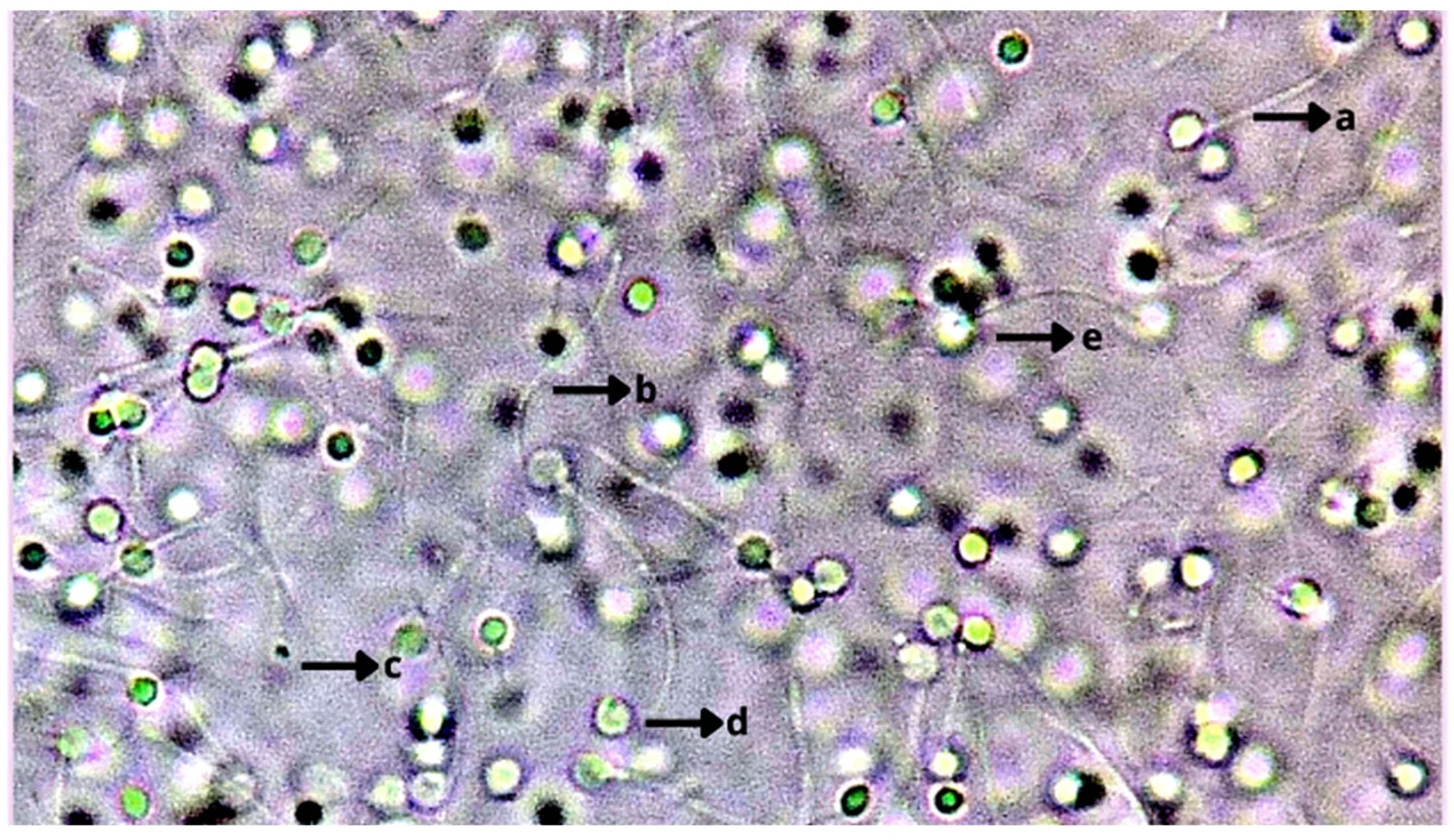
Spermatozoa of Javaen barb (*Systomus rubripinnis*) cryopreserved with basil leaf extract (BLE), stained with Giemsa for morphological assessment. Label (a) indicates normal spermatozoa, (b) tail defect, (c) small head, (d) large head, and (e) coiled tail. Observed under a light microscope (CH30, Olympus, Tokyo, Japan) at 40× objective magnification (400× total).

Post-thaw sperm quality parameters of Javaen barb (*Systomus rubripinnis*) following cryopreservation under BLE supplementation at concentrations ranging from 1% to 4% are summarized in Table 2; representative micrographs of sperm viability and morphology under BLE supplementation are shown in Figure 1 and Figure 2. The 1% BLE treatment demonstrated the highest protective efficacy, yielding peak post-thaw motility (85.00 ± 0.88%), the longest duration of motility (7.14 ± 0.38 min), and the highest cell viability (82.00 ± 1.86%) among all experimental groups, significantly outperforming the antioxidant-free control (65.00 ± 2.31% motility, 4.56 ± 0.33 min duration, and 68.00 ± 2.42% viability; *p* < 0.05). Additionally, the 1% BLE treatment minimized structural damage, resulting in the lowest spermatozoa abnormality rate (18.26 ± 0.16%) compared to the control (23.54 ± 0.28%). The highest fertilization rate was recorded at 2% BLE (92.60 ± 0.57%), followed by 3% (91.41 ± 0.59%) and 1% (88.45 ± 2.72%), while the lowest among the treatments occurred at 4% (82.24 ± 1.36%). Motility, duration, and viability decreased progressively with increasing BLE concentration; fertilization capacity also decreased at the highest BLE dosage.

### 3.3. Post-Thaw Sperm Quality Under Noni Fruit Extract (NFE) Supplementation

**Figure 3:**
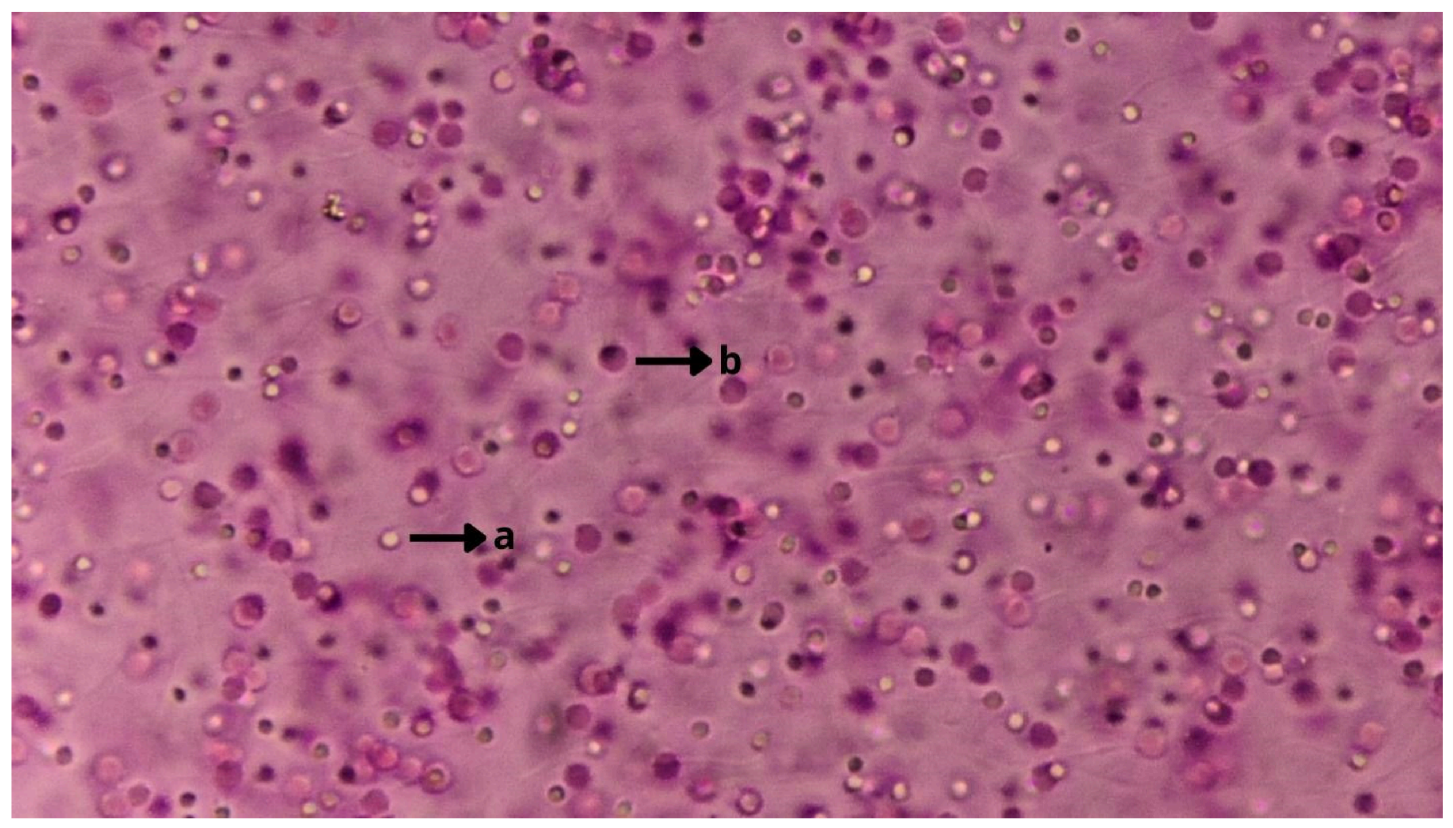
Spermatozoa of Javaen barb (*Systomus rubripinnis*) cryopreserved with noni fruit extract (NFE), stained with eosin-Y for viability assessment. Label (a) indicates viable spermatozoa and label (b) non-viable spermatozoa. Observed under a light microscope (CH30, Olympus, Tokyo, Japan) at 40× objective magnification (400× total).

**Figure 4:**
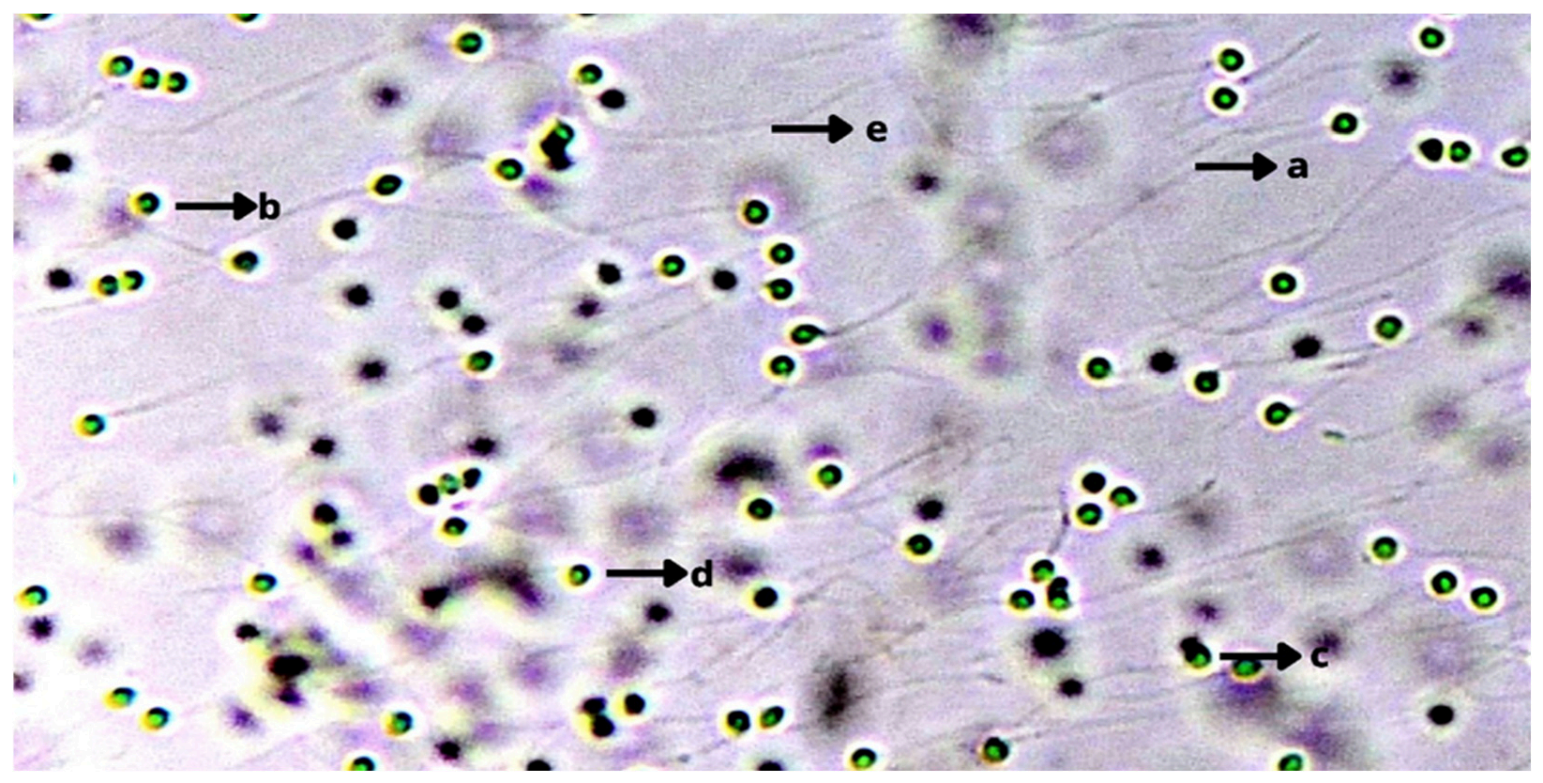
Spermatozoa of Javaen barb (*Systomus rubripinnis*) cryopreserved with noni fruit extract (NFE), stained with Giemsa for morphological assessment. Label (a) indicates normal spermatozoa, (b) tail defect, (c) small head, (d) large head, and (e) coiled tail. Observed under a light microscope (CH30, Olympus, Tokyo, Japan) at 40× objective magnification (400× total).

The effects of noni fruit extract (NFE) supplementation at concentrations ranging from 0.5% to 1.5% on the post-thaw sperm quality parameters of Javaen barb (*Systomus rubripinnis*) are summarized in Table 3; representative micrographs of sperm viability and morphology under NFE supplementation are shown in Figure 3 and Figure 4. Supplementation of NFE demonstrated a distinct dose-dependent response, with the 1% NFE treatment establishing the best-performing protective concentration across major structural and kinetic indicators. Specifically, the 1% NFE group yielded the significantly highest post-thaw motility (81.00 ± 3.28%), the longest duration of motility (5.41 ± 0.28 min), and peak cell viability (71.00 ± 3.51%) compared to both the lower and higher concentration groups (*p* < 0.05). This best-performing treatment also significantly minimized cellular deformities, recording the lowest spermatozoa abnormality rate (8.37 ± 0.38%) among all experimental and control groups. In contrast, the antioxidant-free control group exhibited baseline values of 65.00 ± 2.90% for motility, 4.56 ± 0.26 min for duration, 68.00 ± 3.28% for viability, and 15.35 ± 0.86% for abnormality. Beyond the 1% threshold, higher concentrations induced a sharp physiological decline; at 1.5% NFE, motility and viability dropped significantly to 63.67 ± 4.03% and 54.33 ± 4.51%, respectively, while the duration of motility was severely restricted to 1.81 ± 0.15 min (*p* < 0.05). Among all NFE concentrations, the 1% treatment yielded the highest motility (81.00 ± 3.28%), longest duration (5.41 ± 0.28 min), highest viability (71.00 ± 3.51%), and lowest abnormality (8.37 ± 0.38%), indicating the strongest protective activity at this concentration. The 1.25% NFE treatment produced the highest fertilization rate (90.30 ± 1.91%), followed by 1% (89.60 ± 1.85%) and 1.5% (89.50 ± 0.88%).

### 3.4. Post-Thaw Sperm Quality Under Moringa Leaf Extract (MLE) Supplementation

**Figure 5:**
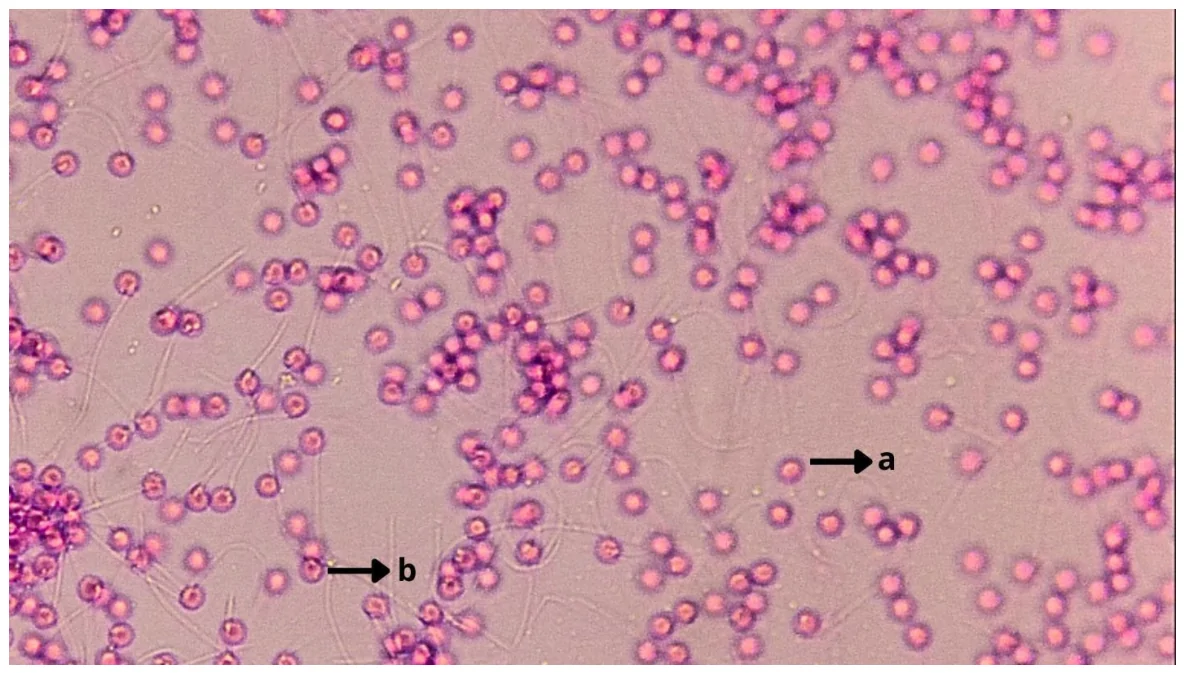
Spermatozoa of Javaen barb (*Systomus rubripinnis*) cryopreserved with moringa leaf extract (MLE), stained with eosin-Y for viability assessment. Label (a) indicates viable spermatozoa and label (b) non-viable spermatozoa. Observed under a light microscope (CH30, Olympus, Tokyo, Japan) at 40× objective magnification (400× total).

**Figure 6:**
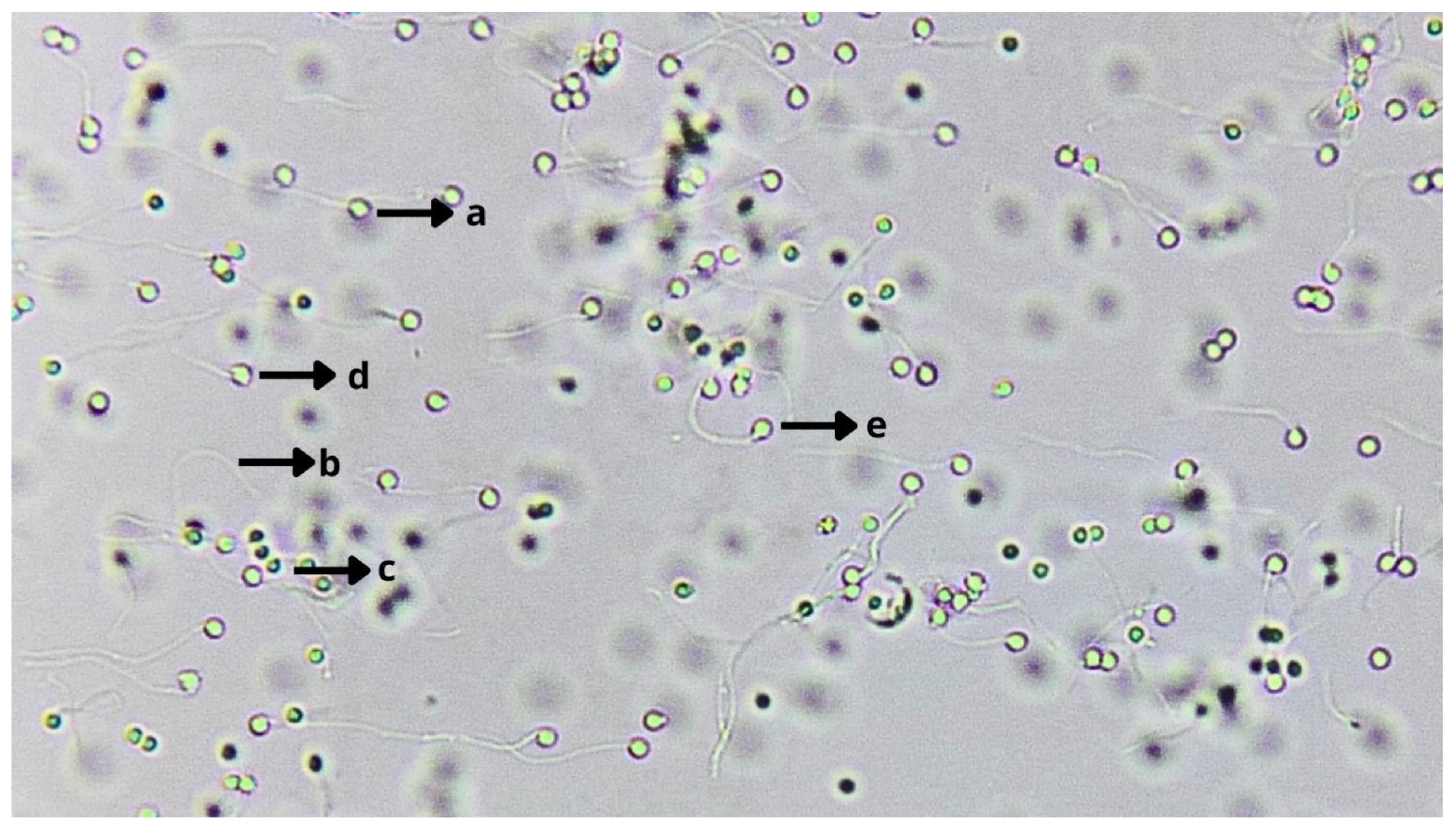
Spermatozoa of Javaen barb (*Systomus rubripinnis*) cryopreserved with moringa leaf extract (MLE), stained with Giemsa for morphological assessment. Label (a) indicates normal spermatozoa, (b) tail defect, (c) small head, (d) large head, and (e) coiled tail. Observed under a light microscope (CH30, Olympus, Tokyo, Japan) at 40× objective magnification (400× total).

The post-thaw sperm quality parameters of Javaen barb (*Systomus rubripinnis*) subjected to cryopreservation under moringa leaf extract (MLE) supplementation at concentrations from 0.5% to 1.5% are presented in Table 4; representative micrographs of sperm viability and morphology under MLE supplementation are shown in Figure 5 and Figure 6. The experimental groups exhibited a distinct biphasic, dose-dependent response, with the 1% MLE treatment demonstrating the highest cryoprotective efficiency across structural and kinetic markers. Specifically, the 1% MLE group yielded the significantly highest post-thaw motility (79.00 ± 1.39%), the longest duration of motility (5.96 ± 0.20 min), and peak cell viability (63.33 ± 3.02%) among all supplemented and control groups (*p* < 0.05). Furthermore, the 1% MLE treatment remarkably mitigated morphological damage, reducing the spermatozoa abnormality rate to its lowest point (7.57 ± 0.39%). In comparison, the antioxidant-free control group (0% MLE) recorded baseline values of 65.00 ± 2.32% for motility, 4.56 ± 0.33 min for duration, 68.00 ± 2.42% for viability, and 23.54 ± 0.28% for abnormality. Beyond the 1% threshold, higher concentrations of the extract induced a severe reversal of the protective effect; at a 1.5% MLE concentration, spermatozoa abnormality rates rose markedly to 20.07 ± 0.15%, while motility and duration dropped significantly to 68.43 ± 1.48% and 4.10 ± 0.24 min, respectively. In terms of reproductive functionality, the maximum fertilization capacity was achieved at the 1% MLE treatment group, reaching a peak of 87.82 ± 7.85%. This was followed in descending mathematical order by the 1.5% MLE concentration (81.19 ± 2.23%) and the 1.25% MLE concentration (80.38 ± 3.95%).

### 3.5. Post-Thaw Sperm Quality Under Honey–Egg Yolk (HEY) Supplementation

**Figure 7:**
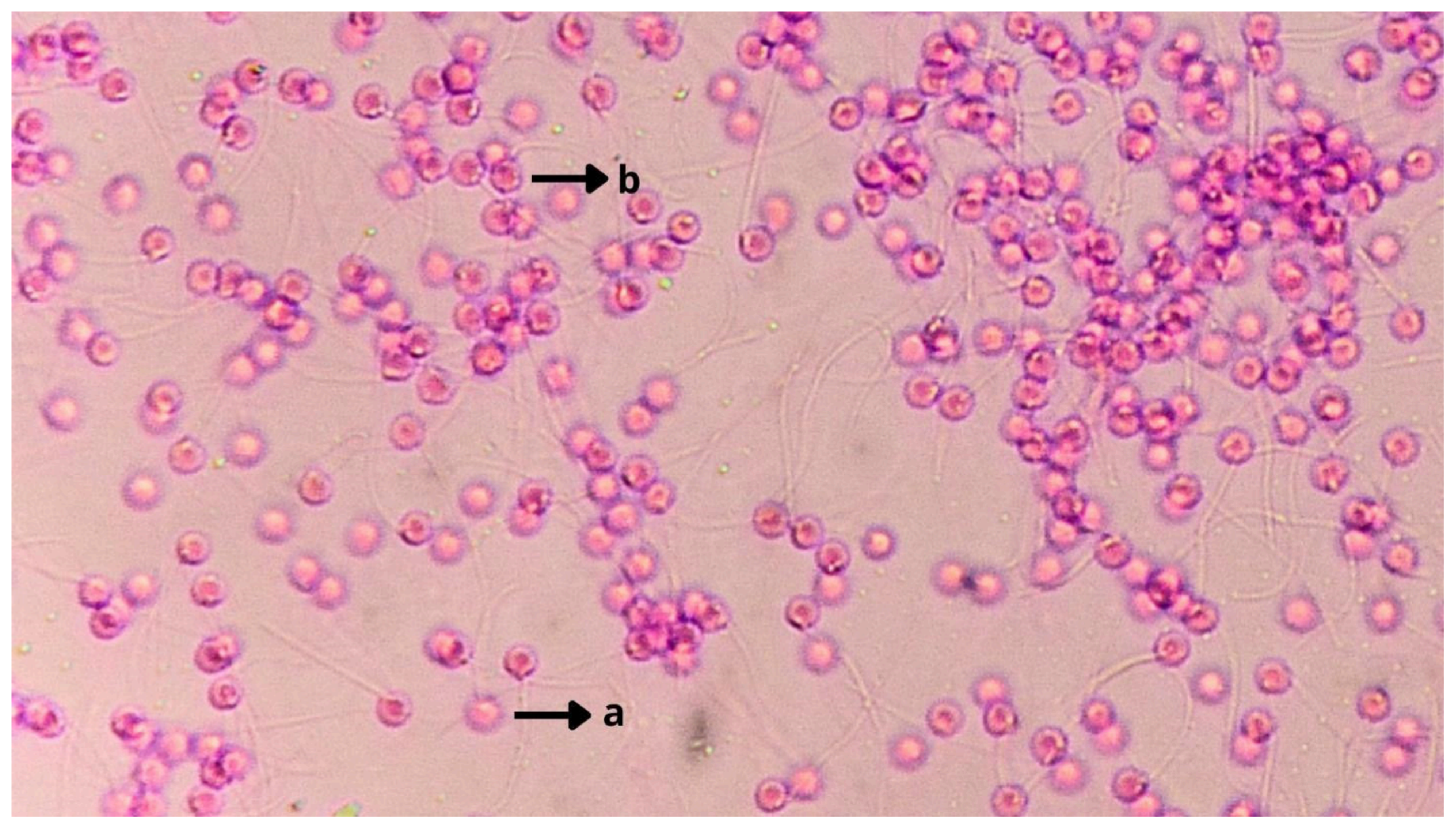
Spermatozoa of Javaen barb (*Systomus rubripinnis*) cryopreserved with honey–egg yolk (HEY), stained with eosin-Y for viability assessment. Label (a) indicates viable spermatozoa and label (b) non-viable spermatozoa. Observed under a light microscope (CH30, Olympus, Tokyo, Japan) at 40× objective magnification (400× total).

**Figure 8:**
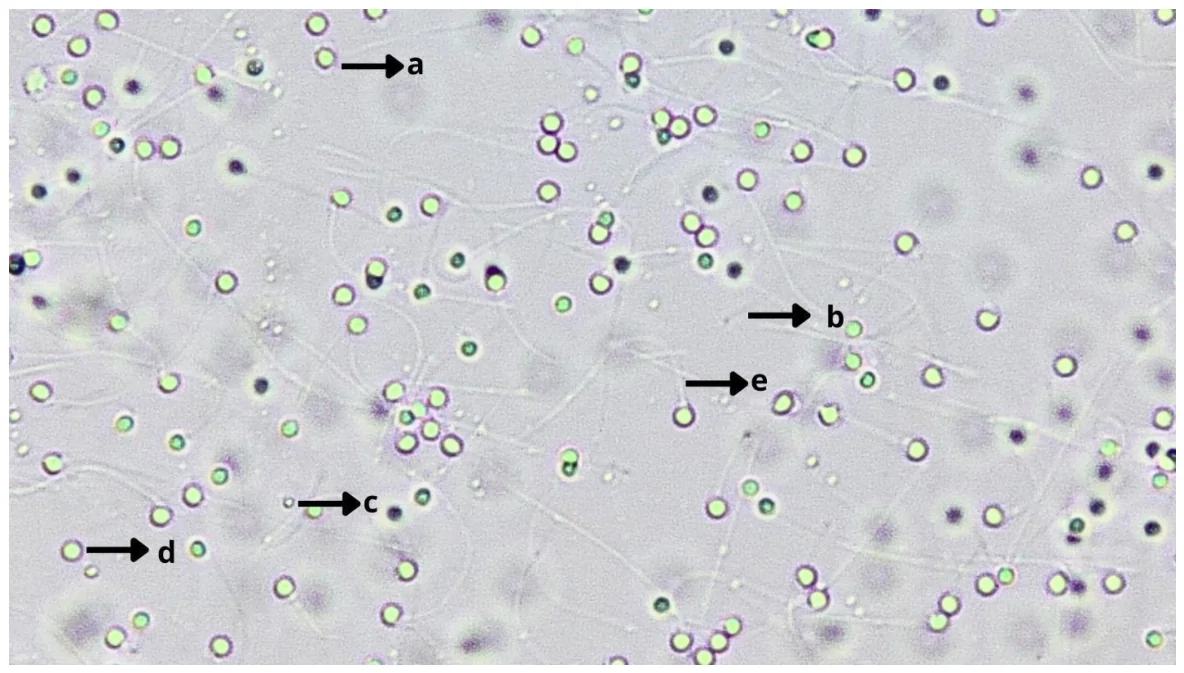
Spermatozoa of Javaen barb (*Systomus rubripinnis*) cryopreserved with honey–egg yolk (HEY), stained with Giemsa for morphological assessment. Label (a) indicates normal spermatozoa, (b) tail defect, (c) small head, (d) large head, and (e) coiled tail. Observed under a light microscope (CH30, Olympus, Tokyo, Japan) at 40× objective magnification (400× total).

The post-thaw sperm quality parameters of Javaen barb (*Systomus rubripinnis*) following cryopreservation under honey–egg yolk (HEY) supplementation–-comprising varying concentrations of honey (1.0% to 2.0%) combined with a fixed 5% egg yolk (EY) matrix–-are detailed in Table 5; representative micrographs of sperm viability and morphology under HEY supplementation are shown in Figure 7 and Figure 8. The experimental groups demonstrated a highly structured protective threshold, with the Honey 1.25% + EY 5% combination establishing the strongest cryoprotective efficiency for physiological and kinetic recovery. This formulation yielded peak post-thaw motility (74.00 ± 3.02% ^a^), the longest duration of sperm motility (5.56 ± 0.39 min ^a^), and the highest cell viability (70.33 ± 2.87% ^a^) across all treatments, while simultaneously minimizing morphological defects to a baseline low abnormality rate of 6.77 ± 0.47% ^d^. In comparison, the standard antioxidant-free control group exhibited baseline values of 65.00 ± 2.31% ^c^ for motility, 4.56 ± 0.33 min ^a^ for duration, 68.00 ± 2.42% ^a^ for viability, and 23.54 ± 0.28% ^c^ for morphological abnormality. In terms of reproductive efficacy, the absolute maximum fertilization capacity was achieved at the highest concentration of Honey 1.25% + EY 5%, reaching a peak rate of 87.89 ± 1.78% ^a^. This was followed in descending mathematical order by the Honey 1.75% + EY 5% treatment group (78.17 ± 0.34% ^b^), the Honey 1.5% + EY 5% group (76.64 ± 1.52% ^b^), and the Honey 2.0% + EY 5% group (75.84 ± 1.50% ^c^).

### 3.6. Post-Thaw Sperm Quality Under Sambiloto Leaf Extract (SLE) Supplementation

**Figure 9:**
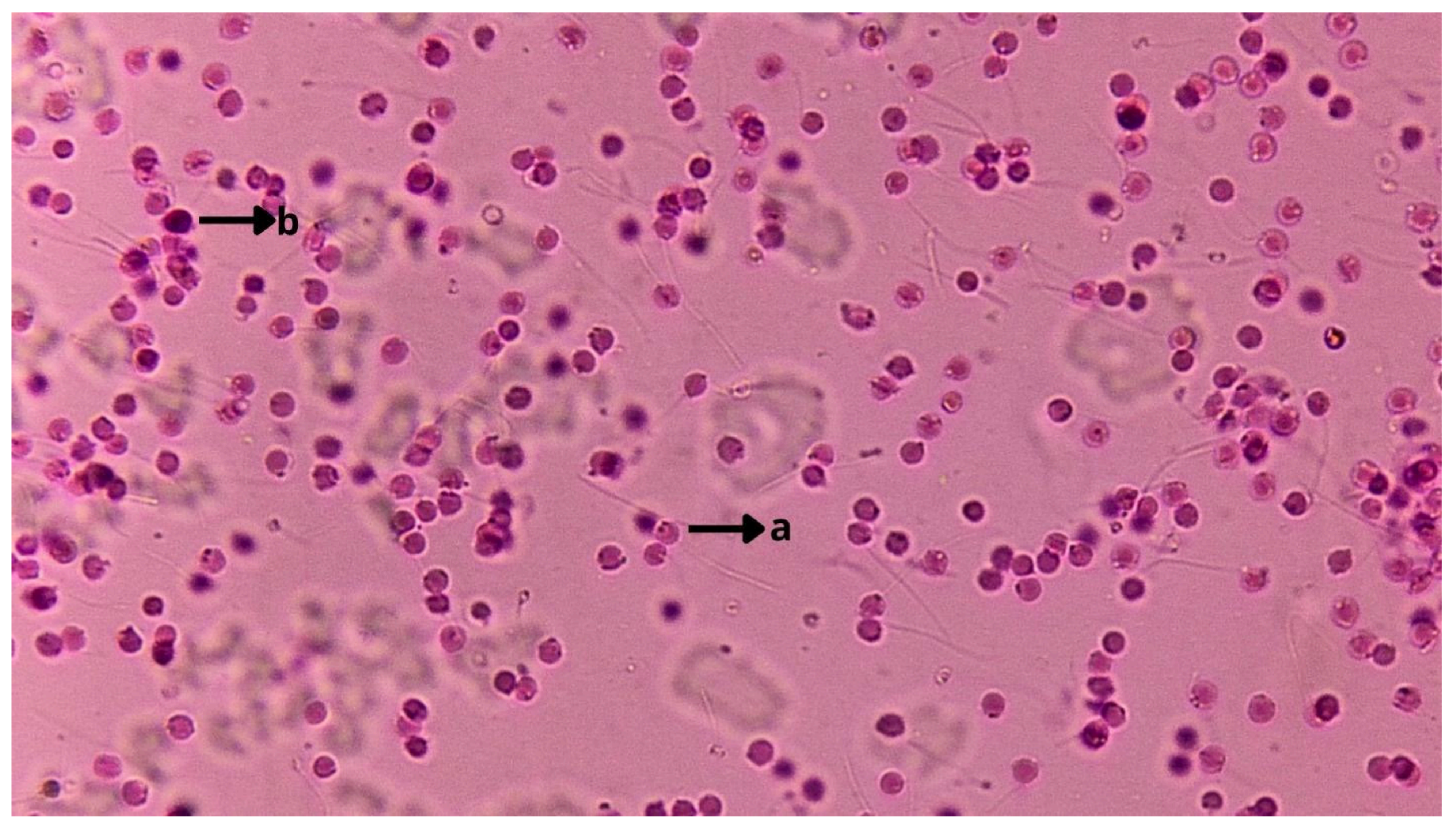
Spermatozoa of Javaen barb (*Systomus rubripinnis*) cryopreserved with sambiloto leaf extract (SLE), stained with eosin-Y for viability assessment. Label (a) indicates viable spermatozoa and label (b) non-viable spermatozoa. Observed under a light microscope (CH30, Olympus, Tokyo, Japan) at 40× objective magnification (400× total).

**Figure 10:**
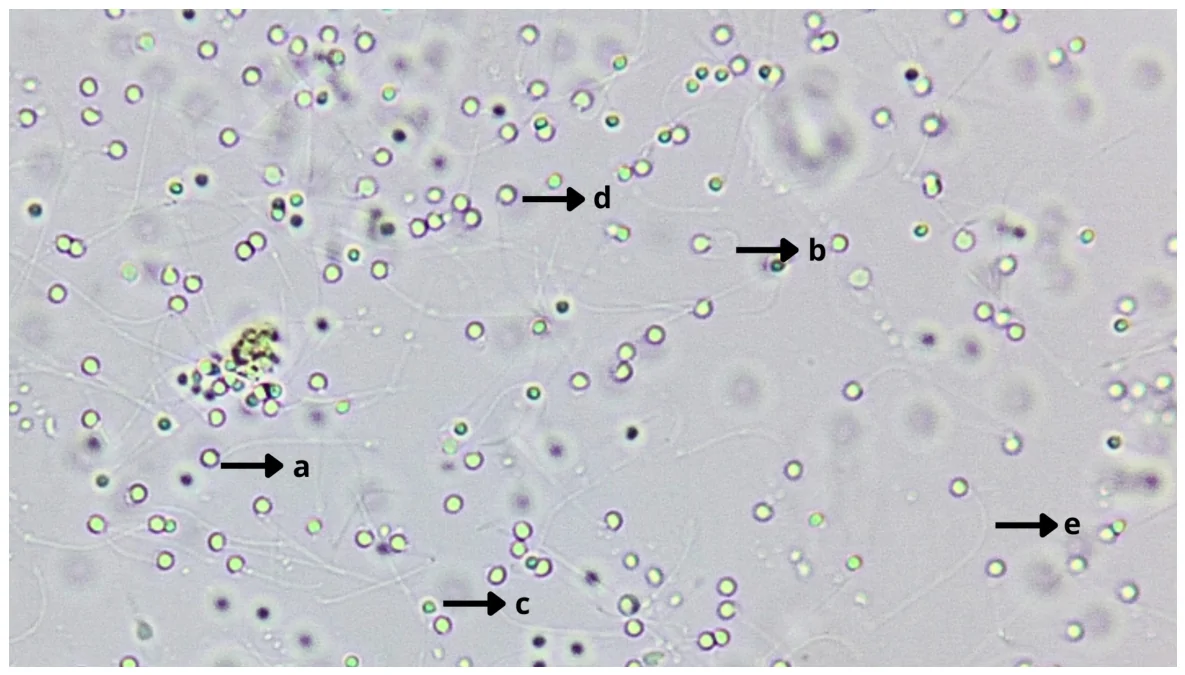
Spermatozoa of Javaen barb (*Systomus rubripinnis*) cryopreserved with sambiloto leaf extract (SLE), stained with Giemsa for morphological assessment. Label (a) indicates normal spermatozoa, (b) tail defect, (c) small head, (d) large head, and (e) coiled tail. Observed under a light microscope (CH30, Olympus, Tokyo, Japan) at 40× objective magnification (400× total).

The post-thaw sperm quality parameters of Javaen barb (*Systomus rubripinnis*) following cryopreservation under sambiloto leaf extract (SLE) supplementation at concentrations ranging from 6% to 9% are presented in Table 6; representative micrographs of sperm viability and morphology under SLE supplementation are shown in Figure 9 and Figure 10. The experimental groups demonstrated unique kinetic resilience, with the 7% SLE treatment yielding peak post-thaw motility (81.00 ± 3.18% ^a^), while the 6% SLE formulation produced the highest descriptive cell viability (71.33 ± 2.50% ^a^). Morphological integrity was maximally preserved at the 7% SLE concentration, which minimized cellular defects to a baseline low abnormality rate of 11.61 ± 0.18% ^a^, outperforming the 6% SLE treatment (12.70 ± 0.15% ^c^). Notably, all SLE-supplemented groups yielded significantly longer post-thaw motility durations (7.54 ± 0.34 min ^a^ to 8.15 ± 0.30 min ^a^) when compared directly to the antioxidant-free control group (4.56 ± 0.29 min ^b^; *p* < 0.05). This exceptional extension of kinetic longevity represents the highest duration values recorded across all botanical antioxidant treatments evaluated in this study.

In terms of reproductive functionality, fertilization rates under SLE supplementation remained uniformly high across the entire tested dose range, extending from 80.05 ± 2.28% ^b^ at 6% SLE to a maximum of 89.64 ± 0.54% ^a^ at 7% SLE. Beyond this best-performing concentration, a progressive reduction was observed, with the fertilization capacity dropping to 82.83 ± 0.44% ^b^ at 8% SLE and reaching its minimum at 9% SLE (76.10 ± 0.56% ^c^). Crucially, all SLE treatments maintained a statistically significant reproductive superiority over the baseline control group, which collapsed to a minimal fertilization rate of 70.08 ± 3.82% ^c^ (*p* < 0.05).

### 3.7. ATP Bioluminescence (Relative Luminescence Intensity)

**Figure 11:**
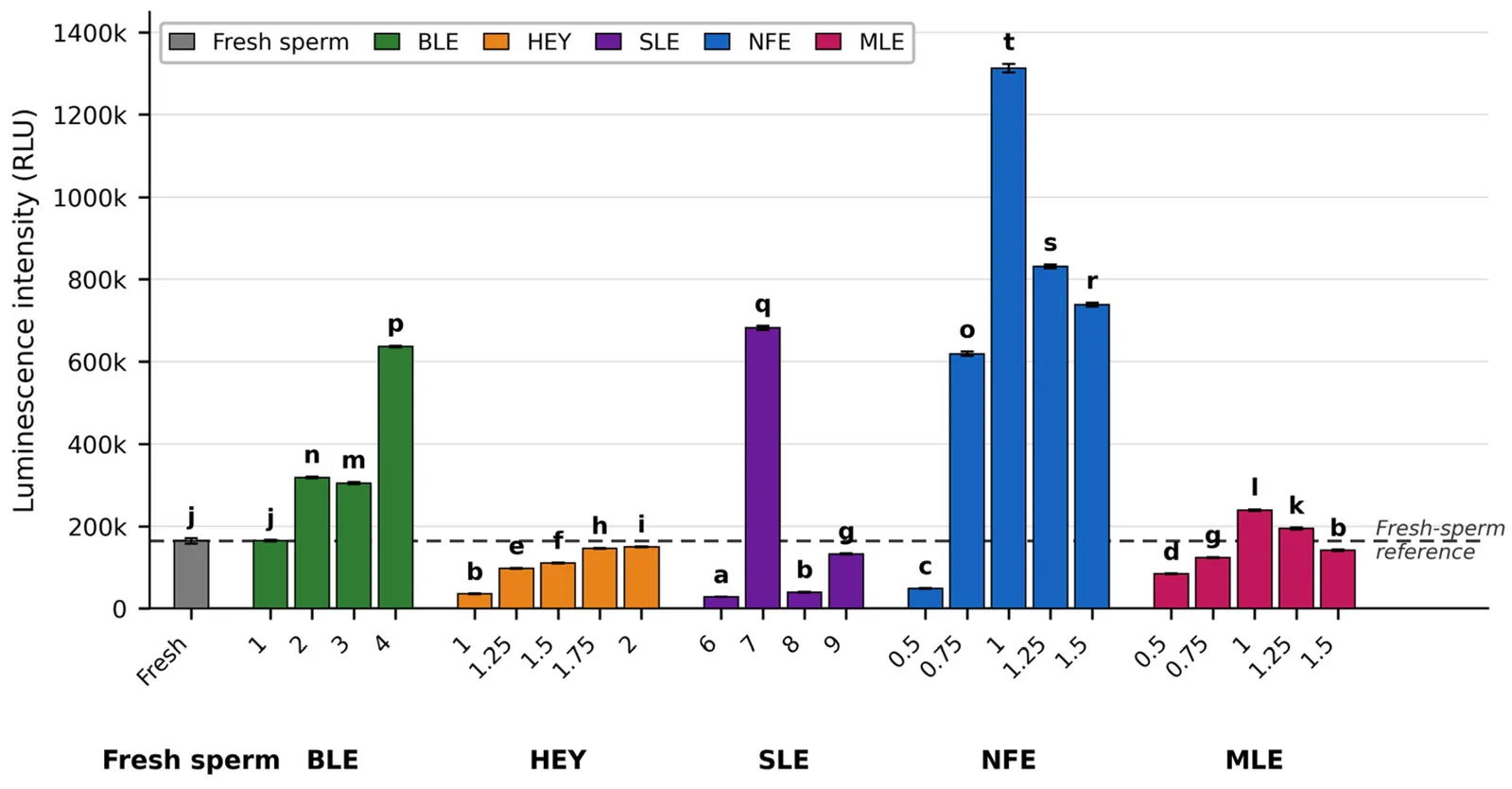
Post-thaw sperm luminescence intensity (RLU; mean ± SD, *n* = 3) of *Systomus rubripinnis* spermatozoa across all 24 treatments, grouped by natural antioxidant (BLE, HEY, SLE, NFE, MLE) and ordered by ascending dose within each group. Bars are means, error bars are standard deviations, and letters above bars denote statistical groupings (Duncan’s multiple range test, *p* < 0.05); bars not sharing a letter differ significantly. The dashed horizontal line marks the fresh-sperm reference (164,087 RLU).

Spermatozoa intracellular adenosine triphosphate (ATP) levels post-thaw were evaluated using a firefly luciferin–luciferase bioluminescence reaction, with light output quantified as relative light units (RLU) across 24 distinct cryopreservation formulations (Figure 11). Prior to statistical testing, the homogeneity of variances was successfully validated via Levene’s test (*p* > 0.05). One-way analysis of variance (ANOVA) revealed highly significant differences in luminescence intensity among the experimental matrices (F = 16.288; *p* < 0.001), a finding robustly corroborated by Welch’s heteroscedastic ANOVA (*p* < 0.001).

Post-thaw bioluminescence profiles exhibited highly differentiated, treatment-specific patterns across the five natural antioxidant series, with values ranging from a minimum of 28,328 ± 249 RLU under the 6% sambiloto leaf extract treatment (SLE 6) to an absolute peak of 1,312,703 ± 10,562 RLU at the 1% noni fruit extract concentration (NFE 1). By comparison, the baseline fresh-sperm reference was established at 164,087 ± 6243 RLU (indicated by the horizontal dashed baseline in Figure 11).

The honey–egg yolk (HEY) and moringa leaf extract (MLE) series, alongside the lower dose groups of the SLE and NFE series, maintained steady metabolic profiles that clustered near or slightly above the fresh-sperm baseline. Within the MLE treatments, a distinct dose-dependent rise culminated in a localized peak at MLE 1 (239,139 RLU; representing 146% of the fresh-sperm baseline) before undergoing a progressive decline beyond the best-performing dose. Conversely, the higher-dose basil leaf extract (BLE) and NFE treatment groups, together with the anomalous 7% sambiloto leaf extract group (SLE 7), exhibited a massive metabolic upregulation, generating luminescence values markedly superior to the fresh-sperm control. The SLE series displayed a striking non-monotonic, biphasic curve, where the SLE 7 treatment diverged sharply from its immediate structural neighboring doses (SLE 6 and SLE 8) to register a localized surge in bioluminescence output, confirming that distinct antioxidant inclusions fundamentally modulate post-thaw mitochondrial energy mechanics.

## 4. Discussion

### 4.1. Effect of Basil Leaf Extract (BLE) on Post-Thaw Sperm Quality

The supplementation of BLE at 1% yielded the highest post-thaw sperm quality across all evaluated parameters, with motility of 85.00 ± 0.88%, duration of motility of 7.14 ± 0.38 min, viability of 82.00 ± 1.86%, and a fertilization rate of 88.45 ± 2.72%. These results suggest a dose-dependent protective effect, with quality indicators declining progressively at 2%, 3%, and 4% concentrations. The superior performance observed at 1% BLE is attributable to the rich bioactive composition of *Ocimum basilicum*, which includes polyphenols, flavonoids (eugenol, linalool, and quercetin), and other phenolic compounds with well-documented antioxidant activity [25]. The strongest antioxidant activity among all tested extracts (IC_50_ = 134.06 ppm) further supports the efficacy of BLE at low concentrations.

These bioactive compounds exert their protective effects primarily by scavenging reactive oxygen species generated during the freeze–thaw cycle, thereby preventing lipid peroxidation of the sperm plasma membrane and maintaining membrane fluidity essential for motility and fertilization [24]. Basil leaf extract has previously been shown to support spermatogenesis and sperm structural integrity in mammalian models. Khaki et al. [24] demonstrated that basil supplementation significantly improved spermatogenic cell architecture and testicular function in rats, effects attributed to the antioxidant and anti-inflammatory activities of its constituent phenolics. Kosari et al. [25] similarly reported improvements in reproductive hormones and semen parameters in Zandi lambs supplemented with dietary basil. The present study is, to our knowledge, the first to document a beneficial effect of basil leaf extract in a fish sperm cryopreservation system. According to Banusiandana et al. [32] the addition of 1% basil leaf extract to a Tris-egg yolk extender was effective in maintaining sperm motility and plasma membrane integrity of Sapudi ram spermatozoa stored at room temperature for up to three hours. Vui et al. [33] stated that *Ocimum basilicum* essential oils affects canine sperm quality in a concentration-dependent manner, with 20 µg/mL being the most effective concentration to reduce oxidative stress and enhance sperm quality during cryopreservation.

A post-thaw motility of 85% represents a comparatively high outcome relative to values reported in other fish cryopreservation studies. For instance, Ali et al. [34] reported post-thaw motility of 79.33 ± 5.13% in Rita catfish using Alsever’s solution with 10% DMSO. In another cyprinid, *Microphysogobio rapidus*, the best post-thaw motility achieved was 59.31 ± 6.57%, using 10% methanol and Ringer’s solution [9]. The superior motility observed with 1% BLE suggests effective mitigation of cryodamage through membrane stabilization and preservation of mitochondrial integrity. At concentrations exceeding 1%, the increased polyphenol load may have induced prooxidant effects or membrane toxicity, consistent with the biphasic dose–response behavior characteristic of many polyphenolic antioxidants [7]. Fertilization rates under BLE treatment were consistently high across all concentrations (82.24–92.60%) and significantly exceeded the control (70.08 ± 3.82%; ANOVA F = 17.41, *p* < 0.001). The highest fertilization rate was recorded at 2% BLE (92.60 ± 0.57%), followed by 3% (91.41 ± 0.59%) and 1% (88.45 ± 2.72%), while the lowest among treatments occurred at 4% (82.24 ± 1.36%). Although motility, duration, and viability declined progressively with increasing BLE concentration, fertilization capacity remained high throughout the tested range.

### 4.2. Effect of Noni Fruit Extract (NFE) on Post-Thaw Sperm Quality

Noni fruit extract at 1% produced the best overall sperm quality profile, with motility of 81.00 ± 3.28%, duration of motility of 5.41 ± 0.28 min, viability of 71.00 ± 3.51%, and the lowest sperm abnormality rate (8.37 ± 0.38%) among all NFE concentrations. These outcomes are consistent with the well-characterized antioxidant phytochemical profile of *Morinda citrifolia*, which includes flavonoids (kaempferol, rutin), iridoids (asperulosidic acid, deacetylasperulosidic acid), and coumarins (scopoletin), all of which exhibit potent free radical scavenging capacity [26]. At 1.25% and 1.5%, the sharp decline in motility duration (2.36 and 1.81 min, respectively) suggests a prooxidant shift at higher NFE concentrations, consistent with the biphasic behavior of polyphenolic antioxidants reported by Kowalczyk [7]. According to Thasmi et al. [35], giving of noni fruit extract significantly affected (*p* < 0.05) on the percentage of motility spermatozoa of boer goat after refrigeration. The allotment of 20% *Morinda citrifolia* L. extracts in the Tris-egg yolk medium was able to maintain the goat’s sperm morphology at −80 °C storage for 24 h [36]. The aqueous extract of noni maintained the function and integrity of the sheep sperm plasma membrane [37]. The DPPH assay yielded a mean IC_50_ of 422.86 ppm for NFE, indicating moderate antioxidant activity. Despite this, the 1% NFE treatment produced a notably low sperm abnormality rate, suggesting that noni-derived compounds may exert protective effects specifically against structural sperm defects through enzymatic upregulation mechanisms rather than direct radical scavenging alone. Hou et al. [26] reported that noni-derived constituents upregulate endogenous antioxidant enzymes, including SOD, CAT, and GPx, which collectively attenuate oxidative damage to cellular membranes and nuclear structures. This enzymatic upregulation may explain the reduced incidence of morphological sperm abnormalities observed at 1%.

### 4.3. Effect of Moringa Leaf Extract (MLE) on Post-Thaw Sperm Quality

Moringa leaf extract at 1% yielded the highest motility (79.00 ± 1.39%), longest duration (5.96 ± 0.20 min), and lowest abnormality (7.57 ± 0.39%) among all MLE treatments. *Moringa oleifera* leaves are a rich source of phenols, flavonoids, proanthocyanidins, vitamins C and E, carotene, zinc, and selenium, all compounds known to mitigate oxidative stress in reproductive cells [27,29]. The multi-target antioxidant mechanism of MLE is well-supported by a growing body of literature demonstrating improvements in post-thaw sperm quality across multiple animal species [28]. Shokry et al. [38] indicated that inclusion of MOLE to semen extender improved the quality and fertility of the post-thawed buffalo bulls’ semen through enhancing the activities of the antioxidant enzyme system and decreasing cryodamage of the buffalo bull spermatozoa. The supplementation with MOLE-200 significantly improved (*p* < 0.05) semen concentration, total motility, progressive motility, and sperm membrane integrity of broiler breeder roosters’ sperm [39]. According to Authaida et al. [40], 1 mg/mL MOLE significantly enhanced total sperm motility, progressive sperm motility, sperm viability, and sperm plasma of Native Thai Bulls’ sperm.

The protective mechanism of MLE in cryopreserved sperm is primarily attributed to the suppression of lipid peroxidation in the sperm plasma membrane, which is enriched with polyunsaturated fatty acids particularly vulnerable to ROS-mediated damage during freezing and thawing [29]. Kamel et al. [28] reported that *Moringa oleifera* leaf extract at 50 mg/100 mL significantly improved sperm motility, viability, acrosomal integrity, and plasma membrane integrity while reducing lipid peroxidation in buck sperm. El-Seadawy et al. [27] similarly demonstrated improved antioxidant capacity in frozen–thawed ram sperm. The present study extends these findings to a teleost fish species for the first time.

At 1.5% MLE, a notable increase in abnormality (20.07 ± 0.15%) was observed despite relatively high motility, suggesting that excessive moringa supplementation may disrupt membrane homeostasis or induce structural alterations in spermatozoa. This aligns with the principle that antioxidant efficacy is concentration-dependent, with excessive supplementation potentially reversing beneficial effects through interference with endogenous redox signaling pathways [7,8].

### 4.4. Effect of Honey–Egg Yolk (HEY) on Post-Thaw Sperm Quality

The H1.25% + EY5% combination produced the most favorable overall sperm quality profile, with the lowest abnormality (6.77 ± 0.47%) and a high fertilization rate (87.89 ± 1.78%). Honey is a complex biological matrix containing phenolic acids, flavonoids (kaempferol, quercetin, chrysin), enzymes (glucose oxidase, catalase), and sugars, all of which contribute to its antioxidant and cryoprotective properties [23]. Egg yolk provides a rich source of low-density lipoproteins (LDL) that stabilize the sperm plasma membrane during freezing by adsorbing to the membrane surface and reducing ice crystal-induced structural disruption. The synergistic combination of honey-derived antioxidant activity and egg yolk-mediated membrane protection in the HEY formulation produced balanced improvements in sperm functional parameters. These findings are consistent with reports in rabbit sperm cryopreservation, where honey supplementation improved motility at lower concentrations but was associated with morphological changes at higher doses [23]. Pasteurized egg yolk and rosemary honey increased ram semen quality after cryopreservation [41]. This stability is due to the synergistic properties of the HEY matrix. Honey functions as an external cryoprotectant; its high fructose and glucose concentrations create a hypertonic extracellular environment that safely dehydrates the cell before freezing, reducing intracellular ice crystal formation [42]. Simultaneously, low-density lipoproteins (LDLs) within the egg yolk bind directly to the flagellar membrane, replacing lost phospholipids and sealing membrane micro-fissures caused by thermal stress [43]. The combining of simple monosaccharides with a lipid-rich protector provides a buffering capacity that shields the spermatozoa flagella across a broader concentration range.

### 4.5. Effect of Sambiloto Leaf Extract (SLE) on Post-Thaw Sperm Quality

Among all SLE concentrations, the 7% treatment yielded the highest post-thaw motility (81.00 ± 3.18%), while 6% showed the best viability and duration motility. The requirement for substantially higher concentrations of SLE (6–9%) compared to other antioxidants in this study (0.5–4%) directly reflects the notably lower antioxidant activity of *Andrographis paniculata* extract (IC_50_ = 3010.46 ppm), indicating that a higher dose is required to achieve equivalent radical scavenging effects. The primary bioactive compound, andrographolide, is a labdane diterpenoid with anti-inflammatory, antioxidant, and immunomodulatory properties [44].

The most notable finding for SLE was the markedly extended duration of motility across all concentrations (7.54–8.15 min), which exceeded that of all other antioxidant treatment groups in this study and was significantly higher than the control (4.56 min). This suggests that andrographolide and associated bioactive compounds may specifically support sperm energy metabolism or axonemal integrity over time, enabling sustained flagellar activity. This is consistent with reports that andrographolide modulates mitochondrial function and reduces oxidative phosphorylation uncoupling, potentially preserving the ATP substrate required for sustained sperm motility [19]. Importantly, SLE has been studied in the context of mammalian reproductive biology primarily in rodents via systemic oral administration where high doses may exert antifertility effects through androgen suppression [45]. However, the application of SLE as a direct additive to cryopreservation media represents a fundamentally different mode of exposure (topical sperm contact vs. systemic administration), making the in vivo antifertility concerns unlikely to apply in the current context. Allan et al. [46] reported an effect of *Andrographis paniculata* extract on spermatozoa of male Wistar rats.

### 4.6. ATP Bioluminescence and Analytical Considerations

ATP is the principal energy source for flagellar movement in fish spermatozoa and a recognized marker of post-thaw sperm quality and mitochondrial integrity [47,48]. The HEY and MLE series and the lower doses of the SLE and NFE series remained at or near the fresh-sperm reference and followed coherent dose-related trends, with MLE showing a clear peak at MLE 1% before declining, consistent with a beneficial antioxidant effect at moderate concentration. These biologically plausible groups support a modest dose-dependent preservation of sperm energy status by the tested antioxidants.

In contrast, several higher-dose BLE and NFE treatments and SLE 7 yielded luminescence several-fold above the fresh-sperm reference, increasing with dose. Because cryopreservation depletes rather than generates ATP, such values are unlikely to reflect a true rise in spermatozoal ATP and more plausibly arise from interference of the extracts with the luciferin–luciferase reaction, which can be enhanced or quenched by co-present substances in a concentration-dependent manner [49,50]. Plant phenolics are known to inhibit luciferase luminescence [51] and to interfere with optical signals in both directions [52], while extract-derived adenylates and altered assay conditions can affect the signal independently of sperm ATP [53]. The data are therefore reported as relative luminescence intensity (RLU) and interpreted comparatively; extract-only blanks and ATP spike-recovery testing are recommended to confirm the higher values, and the markedly elevated, dose-amplified treatments are regarded as provisional pending matrix-corrected measurement.

### 4.7. Study Limitations

Several limitations should be considered when interpreting these findings. First, spermatozoa were obtained from a single pooled sample of fifteen males collected during one spawning season and from one broodstock population, so inter-individual and seasonal variability could not be assessed; validation across multiple males, seasons, and populations is warranted. Second, the antioxidant extracts were characterized functionally by the DPPH assay rather than by compound-level quantification, and the specific bioactive constituents responsible for the observed cryoprotection were therefore inferred from the literature rather than measured directly. Third, the protective mechanisms were interpreted from post-thaw quality outcomes; direct markers of oxidative damage, such as malondialdehyde, reactive oxygen species, and DNA fragmentation, were not quantified. Fourth, ATP content is reported as relative luminescence units (RLU) and interpreted only comparatively, because many treatment values fell outside the assay calibration range and matrix interference from polyphenols and honey constituents may have inflated the signal; absolute ATP concentrations were therefore not derived. Additionally, the honey–egg yolk treatment combined variable honey concentrations with a fixed 5% egg yolk; because egg yolk itself confers membrane and cryoprotective effects, the present design cannot separate the individual contributions of honey, egg yolk, and their interaction, and dedicated honey-only and egg-yolk-only treatment arms would be required to deconvolve them. Moreover, the osmolality, pH, and viscosity of each final formulation—particularly the high-inclusion SLE (6–9%) and honey-containing treatments—were not individually measured or osmotically matched to the control and may modulate the sperm activation response; physicochemical characterization and osmolality/pH-matched controls are recommended in future studies. Two further characterizations that would strengthen mechanistic interpretation, compound-level chromatographic profiling (HPLC/LC-MS) of the extracts, and instrumental measurement of the osmolality, pH, and viscosity of each formulation were beyond the analytical instrumentation and resources available for this study. In their place, the antioxidant capacity of every extract was characterized functionally by the DPPH assay, and the net biological consequences of each complete formulation, including any differences in osmolality, pH, or viscosity, were captured directly through the measured post-thaw endpoints (motility, duration of motility, viability, abnormality, fertilization rate, and ATP). Compound-level profiling and full physicochemical characterization with osmolality/pH-matched controls are accordingly identified as priorities for future work. Finally, fertilization trials used eggs from a limited number of females, and long-term outcomes such as hatching rate, larval survival, and offspring performance were not evaluated. These aspects define clear priorities for future work.

## 5. Conclusions

The optimal antioxidant concentrations for the cryopreservation of Javaen barb (*Systomus rubripinnis*) sperm were BLE 1%, NFE 1%, MLE 1%, HEY (Honey 1.25% + Egg Yolk 5%), and SLE 7%, with BLE yielding the highest post-thaw motility (85%) and viability (82%). All antioxidant treatments supported high post-thaw fertilization rates (>82%). ATP bioluminescence confirmed that 1% BLE preserved post-thaw sperm energy status equivalent to fresh sperm, consistent with a dose-dependent response. These plant-derived antioxidants offer a practical and scientifically supported approach for improving sperm cryopreservation protocols in Javaen Barb hatcheries and endemic freshwater fish conservation programs. Beyond their antioxidant efficacy, these plant-derived extracts offer clear practical advantages over synthetic alternatives: they are locally abundant, low-cost, biodegradable, and non-toxic, and can be prepared with minimal laboratory infrastructure, making them particularly well suited to community-based and small-scale hatchery programs for the conservation of endemic freshwater species. Among the five systems compared, 1% BLE provided the most effective overall post-thaw protection, whereas SLE was distinctive in sustaining the longest motility duration. Because this study compared a discrete set of concentrations rather than performing a formal optimization, future work applying response-surface or comparable optimization designs is warranted to define the most effective supplementation levels more precisely.

## Figures and Tables

**Table 1:** DPPH radical scavenging activity (IC_50_, ppm) of the four plant extracts evaluated in this study. Values are means ± SE of three replicates (*n* = 3).

No.	Sample	IC_50_ Replicate 1 (ppm)	IC_50_ Replicate 2 (ppm)	Mean IC_50_ (ppm)
1	BLE	134.9189	133.2060	134.06
2	MLE	297.1451	296.9321	297.04
3	NFE	420.9269	424.7854	422.86
4	SLE	3011.2420	3009.6752	3010.46

Lower IC_50_ values indicate stronger antioxidant activity. Honey–egg yolk (HEY) was not evaluated by DPPH because it is a complex biological matrix rather than a pure plant extract. BLE, basil leaf extract; MLE, moringa leaf extract; NFE, noni fruit extract; SLE, sambiloto leaf extract.

**Table 2:** Post-thaw sperm quality of Javaen barb (*Systomus rubripinnis*) cryopreserved with basil leaf extract (BLE) at 1–4%. Values are means ± SEs of three replicates (*n* = 3).

BLE (%)	Motility (%)	Duration (min)	Viability (%)	Abnormality (%)	Fertilization Rate (%)
Control	65.00 ± 2.31 ^c^	4.56 ± 0.33 ^b^	68.00 ± 2.42 ^b^	23.54 ± 0.28 ^c^	70.08 ± 3.82 ^b^
1%	85.00 ± 0.88 ^a^	7.14 ± 0.38 ^a^	82.00 ± 1.86 ^a^	18.26 ± 0.16 ^c^	88.45 ± 2.72 ^a^
2%	73.00 ± 1.84 ^b^	5.35 ± 0.35 ^b^	70.67 ± 1.35 ^b^	23.40 ± 0.22 ^b^	92.60 ± 0.57 ^a^
3%	64.00 ± 1.84 ^c^	4.10 ± 0.24 ^c^	61.67 ± 2.83 ^c^	24.90 ± 0.27 ^b^	91.41 ± 0.59 ^a^
4%	58.00 ± 3.71 ^d^	3.36 ± 0.32 ^d^	55.67 ± 3.17 ^d^	27.61 ± 0.26 ^a^	82.24 ± 1.36 ^a^

Different superscript letters within a column indicate significant differences (Duncan’s multiple range test, *p* < 0.05).

**Table 3:** Post-thaw sperm quality of Javaen barb (*Systomus rubripinnis*) cryopreserved with noni fruit extract (NFE) at 0.5–1.5%. Values are means ± SEs of three replicates (*n* = 3).

NFE (%)	Motility (%)	Duration (min)	Viability (%)	Abnormality (%)	Fertilization Rate (%)
Control	65.00 ± 2.31 ^c^	4.56 ± 0.33 ^b^	68.00 ± 2.42 ^a^	23.54 ± 0.28 ^a^	70.08 ± 3.82 ^b^
0.5%	61.00 ± 2.85 ^d^	2.84 ± 0.19 ^d^	56.67 ± 2.67 ^d^	16.87 ± 0.55 ^b^	73.60 ± 0.29 ^b^
0.75%	66.00 ± 1.35 ^c^	3.01 ± 0.35 ^c^	60.33 ± 3.91 ^c^	16.33 ± 1.40 ^b^	76.20 ± 1.98 ^b^
1%	81.00 ± 3.28 ^a^	5.41 ± 0.28 ^a^	71.00 ± 3.51 ^a^	8.37 ± 0.38 ^d^	89.60 ± 1.85 ^a^
1.25%	74.30 ± 1.50 ^b^	2.36 ± 0.30 ^d^	63.67 ± 4.03 ^b^	14.53 ± 0.32 ^c^	90.30 ± 1.91 ^a^
1.5%	75.00 ± 4.36 ^b^	1.81 ± 0.15 ^e^	52.33 ± 1.64 ^e^	20.67 ± 0.51 ^a^	89.50 ± 0.88 ^a^

Different superscript letters within a column indicate significant differences (Duncan’s multiple range test, *p* < 0.05).

**Table 4:** Post-thaw sperm quality of Javaen barb (*Systomus rubripinnis*) cryopreserved with moringa leaf extract (MLE) at 0.5–1.5%. Values are means ± SEs of three replicates (*n* = 3).

MLE (%)	Motility (%)	Duration (min)	Viability (%)	Abnormality (%)	Fertilization Rate (%)
0%	65.00 ± 2.32 ^c^	4.56 ± 0.33 ^b^	68.00 ± 2.42 ^a^	23.54 ± 0.28 ^a^	70.08 ± 3.82 ^c^
0.5%	63.00 ± 3.02 ^c^	3.65 ± 0.25 ^c^	54.33 ± 2.04 ^b^	15.83 ± 0.41 ^b^	71.25 ± 0.63 ^c^
0.75%	67.00 ± 1.45 ^c^	4.40 ± 0.27 ^b^	56.67 ± 3.02 ^b^	12.37 ± 0.38 ^c^	73.82 ± 0.73 ^c^
1%	79.00 ± 1.39 ^a^	5.96 ± 0.20 ^a^	63.33 ± 3.02 ^a^	7.57 ± 0.39 ^d^	87.82 ± 7.85 ^a^
1.25%	72.70 ± 2.17 ^b^	4.35 ± 0.24 ^b^	62.33 ± 1.84 ^a^	13.90 ± 0.53 ^c^	80.38 ± 3.95 ^b^
1.5%	78.00 ± 2.46 ^a^	4.28 ± 0.34 ^b^	60.33 ± 2.34 ^a^	20.07 ± 0.15 ^a^	81.19 ± 2.23 ^b^

Different superscript letters within a column indicate significant differences (Duncan’s multiple range test, *p* < 0.05).

**Table 5:** Post-thaw sperm quality of Javaen barb (*Systomus rubripinnis*) cryopreserved with honey (1.0–2.0%) combined with 5% egg yolk. Values are means ± SEs of three replicates (*n* = 3).

HEY Treatment	Motility (%)	Duration (min)	Viability (%)	Abnormality (%)	Fertilization Rate (%)
Control	65.00 ± 2.31 ^c^	4.56 ± 0.33 ^a^	68.00 ± 2.42 ^a^	23.54 ± 0.28 ^c^	70.08 ± 3.82 ^c^
Honey 1% + EY 5%	71.00 ± 1.35 ^b^	4.81 ± 0.23 ^b^	64.00 ± 2.65 ^b^	13.17 ± 0.58 ^b^	71.04 ± 1.58 ^c^
Honey 1.25% + EY 5%	74.00 ± 3.02 ^a^	5.56 ± 0.39 ^a^	70.33 ± 2.87 ^a^	6.77 ± 0.47 ^d^	87.89 ± 1.78 ^a^
Honey 1.5% + EY 5%	72.00 ± 3.18 ^a^	4.32 ± 0.43 ^c^	64.33 ± 2.14 ^b^	11.30 ± 0.47 ^c^	76.64 ± 1.52 ^b^
Honey 1.75% + EY 5%	70.70 ± 1.84 ^b^	4.23 ± 0.25 ^c^	61.67 ± 2.69 ^c^	16.30 ± 0.43 ^a^	78.17 ± 0.34 ^b^
Honey 2% + EY 5%	69.00 ± 3.00 ^b^	4.07 ± 0.26 ^c^	61.33 ± 1.26 ^c^	22.97 ± 0.57 ^a^	75.84 ± 1.50 ^b^

EY, egg yolk. Different superscript letters within a column indicate significant differences (Duncan’s multiple range test, *p* < 0.05).

**Table 6:** Post-thaw sperm quality of Javaen barb (*Systomus rubripinnis*) cryopreserved with sambiloto leaf extract (SLE) at 6–9%. Values are means ± SEs of three replicates (*n* = 3).

SLE Treatment	Motility (%)	Duration (min)	Viability (%)	Abnormality (%)	Fertilization Rate (%)
0%	65.00 ± 2.22 ^a^	4.56 ± 0.29 ^b^	68.00 ± 2.55 ^a^	17.45 ± 0.27 ^b^	70.08 ± 3.82 ^c^
6%	72.00 ± 1.45 ^a^	8.15 ± 0.30 ^a^	71.33 ± 2.50 ^a^	12.70 ± 0.15 ^d^	80.05 ± 2.28 ^b^
7%	81.00 ± 3.18 ^a^	8.14 ± 0.30 ^a^	68.33 ± 2.67 ^a^	11.61 ± 0.18 ^e^	89.64 ± 0.54 ^a^
8%	77.00 ± 2.01 ^a^	7.85 ± 0.19 ^a^	65.33 ± 1.90 ^a^	13.45 ± 0.18 ^c^	82.83 ± 0.44 ^b^
9%	67.00 ± 1.86 ^a^	7.54 ± 0.34 ^a^	64.67 ± 2.99 ^a^	19.06 ± 0.35 ^a^	78.52 ± 1.44 ^b^

Different superscript letters within a column indicate significant differences (Duncan’s multiple range test, *p* < 0.05).

## Data Availability

The data presented in this study are available on request from the corresponding author.

## Abbreviations

The following abbreviations are used in this manuscript:

ATP	Adenosine triphosphate
BLE	Basil leaf extract (*Ocimum basilicum*)
CCK-8	Cell counting kit-8
DPPH	2,2-diphenyl-1-picrylhydrazyl
HEY	Honey–egg yolk
IC_50_	Half-maximal inhibitory concentration
LDL	Low-density lipoprotein
MLE	Moringa leaf extract (*Moringa oleifera*)
NFE	Noni fruit extract (*Morinda citrifolia*)
PUFA	Polyunsaturated fatty acid
RLU	Relative luminescence unit
ROS	Reactive oxygen species
SLE	Sambiloto leaf extract (*Andrographis paniculata*)
